# Flexibility of orthographic and graphomotor coordination during a handwritten copy task: effect of time pressure

**DOI:** 10.3389/fpsyg.2013.00866

**Published:** 2013-11-22

**Authors:** Solen Sausset, Eric Lambert, Thierry Olive

**Affiliations:** Centre de Recherches sur la Cognition et l'Apprentissage, CNRS and University of PoitiersFrance

**Keywords:** flexibility, orthographic and graphomotor coordination, handwriting, time pressure, syllable processing

## Abstract

The coordination of the various processes involved in language production is a subject of keen debate in writing research. Some authors hold that writing processes can be flexibly coordinated according to task demands, whereas others claim that process coordination is entirely inflexible. For instance, orthographic planning has been shown to be resource-dependent during handwriting, but inflexible in typing, even under time pressure. The present study therefore went one step further in studying flexibility in the coordination of orthographic processing and graphomotor execution, by measuring the impact of time pressure during a handwritten copy task. Orthographic and graphomotor processes were observed via syllable processing. Writers copied out two- and three-syllable words three times in a row, with and without time pressure. Latencies and letter measures at syllable boundaries were analyzed. We hypothesized that if coordination is flexible and varies according to task demands, it should be modified by time pressure, affecting both latency before execution and duration of execution. We therefore predicted that the extent of syllable processing before execution would be reduced under time pressure and, as a consequence, syllable effects during execution would be more salient. Results showed, however, that time pressure interacted neither with syllable number nor with syllable structure. Accordingly, syllable processing appears to remain the same regardless of time pressure. The flexibility of process coordination during handwriting is discussed, as is the operationalization of time pressure constraints.

## Introduction

How language is planned is a central question in psycholinguistic research. For instance, investigating how the different processes involved in spoken language production are coordinated is key to understanding the high fluency and efficiency of the oral language production system. Although sequential and parallel models of language production processes have been developed (e.g., Dell, 1986; Levelt et al., 1999), cascade models appear to describe the time course of language production most accurately (McClelland, 1979; Kempen and Hoenkamp, 1987; Humphreys et al., 1988). Cascade models assume that each segment of language is processed in a sequential or hierarchical order, moving from the central to the peripheral levels of processing (i.e., from content planning to linguistic formulation and articulation). In addition, they assume that different segments of language can be processed in parallel at different levels of representation. One advantage of cascade models is their functional flexibility: some segments (or units) of language are processed just before their articulation, whereas others are planned in advance. Research on advance planning has confirmed that speakers can adapt the coordination of their cognitive processing during language production by modulating the scope of their advance planning (Oppermann et al., 2010; Wagner et al., 2010; Konopka, 2012).

In contrast to oral production, relatively little is known about the coordination of writing processes. Van Galen (1991) proposed a cascade model whereby, as in speech, all the writing processes can be activated simultaneously, but higher-order processes (e.g., ideas planning) always take place ahead of lower-order ones. This model, however, does not describe the mechanisms that allow for flexibility in the way the processes overlap. In the present study, we therefore investigated flexibility in the coordination of processes in written word production by administering a handwriting task with or without time pressure. We reasoned that imposing time pressure during a writing task would increase task demands by modifying the self-paced flow of handwriting, and by so doing would modify the coordination of the orthographic and graphomotor processes.

### Flexibility in coordinating high-level writing processes with transcription

Writing a text requires high- and low-level processes devoted to content planning (selection and organization of ideas), language formulation–or translating (syntactic and lexical choices, spelling), transcription (handwriting or typing), and revision of the text being produced. High-level writing processes cascade onto low-level ones. For example, in adults (but not in children, who rely on low-level, resource-demanding writing processes), the preparation of one text segment takes place concurrently with the transcription of the previous one (Chanquoy et al., 1990). Similarly, adult writers are able to read portions of the text they have already produced while transcribing a new segment (Alamargot et al., 2007). In sum, the concurrent activation of high-level writing processes with motor execution is now well documented, with translating being the most frequently activated process during transcription, followed by planning and revising [for typing, see Alves et al. (2008); for handwriting, see Olive et al. (2009)].

Of major relevance to the present study, researchers have already demonstrated the flexibility of the time course of the high-level planning, translating and revising processes, in relation to the low-level processes engaged in handwriting. For instance, adults who transcribe their text in their own familiar, automatized handwriting can concurrently engage high-level processes. However, when they have to transcribe their text in an unfamiliar style of handwriting, such as uppercase cursive letters, they shift to more sequential coordination of the writing processes (Olive and Kellogg, 2002). Similarly, when adult writers transcribe their text without being able to see what they have just written (i.e., when they are prevented from visually monitoring their handwriting) they also shift to more sequential coordination (Olive and Piolat, 2002). Instead of being engaged in a set and rigid manner, the processes involved in handwriting can therefore be adapted to match the task's specific demands and characteristics.

Flexibility in the coordination of cognitive processing may therefore be modulated by the demands of the writing task, as all the levels of processing require working memory capacity (see Olive, 2004, 2012, for reviews), and compete for a common pool of finite resources (Kellogg, 2001). Accordingly, flexibility in the coordination of writing processes is essential, if writers are to adapt to the changing demands of writing, and we would expect the scope of advance planning to depend on task demands.

### Flexibility in coordinating orthographic processes with transcription

Flexibility in the time course of the writing processes can also be observed in transcription despite the fact that the processes engaged in transcription are automatized to a greater degree than the processes involved in either text planning or revising. At a sublexical level, the coordination of orthographic and graphomotor processes has been shown to vary according to lexical specificity. For instance, Roux et al. (2013) showed that there is an effect of lexicality and spelling regularity on letter duration in a copy task. They showed that when writing words, spelling processes cascade onto execution, but only for first few letters; however, when writing pseudo-words, sublexical processes impact graphomotor processing even during the execution of the final letters. Moreover, as regards words, the overlap between orthographic and graphomotor processes was greater when spelling irregularities were located at the end of the word than when they were located at the beginning of the word. These results are consistent with those of Delattre et al. (2006) who showed that in the case of regular words, spelling processes are completed prior to graphomotor execution, but in the case of irregular words, the spelling processes continue while the word is being written, that is, in parallel with execution.

As well as overlapping with the graphomotor execution of the word in question, the spelling process may also begin while the previous word is still being written (Lambert et al., 2011). In a task requiring several words to be copied at a time, information searches (captured through eye movement recordings) were found to be triggered at different junctures, depending on the word's lexical features. In the case of frequent and regular words, these searches took place in parallel with the execution of the end of the previous word.

The flexibility of cascading processes has also been observed for syllable processing in relation to transcription. The syllable can be regarded as one of the processing units involved in orthographic processing (e.g., Kandel et al., 2006; Lambert et al., 2008). Sausset et al. (2012) asked writers to copy out two- and three-syllable words three times in a row, using four different handwriting modes: lowercase, uppercase, large uppercase, and large uppercase with no visual feedback. Latencies, as well as interletter intervals both at syllable boundaries and before and after those boundaries were analyzed. An effect of syllable number was only observed in the lowercase condition, that is, the least demanding one. However, the duration of the interletter intervals at the syllable boundaries increased relative to intrasyllabic ones when graphomotor constraints increased. Sausset et al. (2012) interpreted these findings as evidence that all the syllables in the words were processed prior to execution in the low graphomotor constraint condition, whereas each syllable was sequentially processed at the syllable boundary in the high graphomotor constraint condition. When individuals use their usual handwriting, therefore, orthographic planning takes place in advance, before the onset of execution. However, when the graphomotor constraints are high, orthographic processing is postponed and takes place during execution, at the syllable boundaries. In sum, the coordination of orthographic and graphomotor processes is flexible and is modulated by handwriting demands.

In Sausset et al.'s (2012) study, the processing demands of handwriting were manipulated by asking participants to copy the target words out in four different output modes, three of which were unfamiliar. One alternative interpretation of Sausset et al.'s findings is thus that, instead of directly affecting the coordination of the underlying processes, the unfamiliar handwriting indirectly affected the time course by modifying the nature of the processing unit (i.e., shifting from syllable to letter processing). In order to study flexibility in writing process coordination without altering the units of processing—as is potentially the case when uppercase handwriting is required—, it is therefore important to allow participants to use their normal handwriting. Furthermore, a different form of constraint is needed—one that allows for a direct comparison of coordination in different constrained conditions.

One way of varying task demands without affecting either the processing unit or the participants' familiarity with the handwriting is time pressure, which is an ecological constraint, as people are frequently required to write under time pressure in the course of their daily lives (e.g., during note-taking; Piolat et al., 2005). Research on speech production has also shown that time pressure results in an overlap between overt articulation and the central processes (Kello et al., 2000). In text writing under time pressure, priority is given to the higher-level writing processes at the expense of handwriting (Brown et al., 1988). In a word production task, Damian and Freeman (2008) examined the effects of a time pressure constraint. In their first experimental study, typists performed a Stroop interference task, in which they had to write the name of a color patch while ignoring the name of a color written on it. The colors of the patches and the color labels were either congruent or incongruent. In their second experiment, the authors used the same task, but the participants also had to cope with a deadline, and were thus under time pressure. Results showed that in both experiments, the color-word interference had a significant effect on latencies and error rates, revealing that some—if not all—orthographic planning takes place in advance. Nevertheless, although latencies were shorter under time pressure, no interference effect was observed on execution durations, prompting the authors to say that no orthographic processing took place during execution, even under time pressure. Accordingly, Damian and Freeman (2008) concluded that there is no flexibility in the coordination of the writing processes.

Although their conclusion is at odds with Sausset et al.'s (2012) findings, differences in output modalities may account for the conflicting conclusions reached in these two studies. Whereas Sausset et al. (2012) asked participants to write the words by hand, Damian and Freeman (2008) asked their participants to type the names of the colors. As handwriting is learned much earlier than typing, and is therefore a more automatized skill, the participants in Damian and Freeman's study were presumably less skilled in word transcription than those in Sausset et al.'s study. Accordingly, the time course and articulation of the processes involved may have differed. The fact remains that the impact of time pressure on the flexibility of the coordination of cognitive processes has never been studied in a written task performed in the participants' usual handwriting.

### The present study

We therefore investigated flexibility in the coordination of orthographic syllable processing and graphomotor processing further, as a function of time pressure. Our main hypothesis, in the light of Sausset et al.'s (2012) results, was that coordination during handwriting is flexible. As in their study, we used a word copy task in which participants had to copy two- and three-syllable words out three times in a row, but solely in their usual handwriting. Selecting handwriting instead of typing ensured that transcription was fully automatized in the adult writers who took part in the study. The multiple copy task was performed in two conditions: participants wrote the words either at their usual handwriting speed or else under time pressure. We predicted that time pressure would modify the way that the orthographic and graphomotor processing of the syllables was coordinated. Where there was a need for speed, we expected syllable processing, which is usually anticipated before graphomotor programming, to be less anticipated and hence to be postponed onto execution.

Following previous studies showing that, with usual handwriting, latency increases with the number of syllables in the word being copied, and that this effect is sensitive to task constraints (Lambert et al., 2008; Sausset et al., 2012), we measured latencies to determine whether the orthographic processing of syllables prior to graphomotor execution is affected by time pressure. We also measured word duration, as in Damian and Freeman (2008). However, because execution is relatively slow with handwritten output, making word duration a rather rough measure of the underlying written processes, we also analyzed measures at the single-letter level at the syllable boundaries. More specifically, we calculated letter stroke duration and trajectory length (e.g., Zesiger et al., 1994; Bogaerts et al., 1996) as well as fluency, represented by the number of velocity peaks per stroke (e.g., Kandel et al., 2009) for a letter before and after the syllable boundary. The latter measures have been shown to be affected by the words' syllabic structure.

If the coordination of orthographic syllable processing and graphomotor processing was flexible, we would observe selective effects of time pressure on intercopy latency and measures at the single-letter level. With time pressure, syllable processing would be reduced prior to execution (i.e., during the latencies), and would be at least partially postponed until execution (i.e., at the syllable boundaries). Accordingly, an effect of the interaction between syllable number and writing condition would be observed on latency. More specifically, syllable number would have a significant effect on latency with usual writing speed, but this effect would be reduced or become non-significant with time pressure. As a consequence, the syllable boundary effect (difference between letters before and after the boundary) would be greater under time pressure because orthographic processing would not have been entirely completed prior to graphomotor execution. Therefore, an interaction between syllable boundary and writing condition would also be observed. By contrast, if the coordination of orthographic and graphomotor processes was inflexible, then no interaction would be observed, either between writing condition and syllable number, or between writing condition and syllable boundary.

## Materials and methods

### Participants

Twenty adults (6 women, 14 men) with at least a BA and who regularly practiced handwriting took part in the experiment on a voluntary basis. All participants were native French speakers and reported having normal or normal-to-corrected vision. Seventeen were right-handed. Their mean age was 23; 4 years (range: 19; 6–30; 3). Each of them signed an informed consent form at the beginning of the experiment.

### Material

The experiment was run using Eye and Pen software (Alamargot et al., 2006) on a PC computer. A graphics tablet (WACOM Intuos_3_ A5 PTZ-631) and a contact inking pen (WACOM Intuos_3_ Ink Pen) were used to record the written data and to present the words to copy. These words were printed in 11-mm Arial font lowercase black letters against a white background. Participants copied the target words on a sheet of paper placed on the tablet. On this sheet were drawn several cues to guide the multiple copy task: to the left, a start box to trigger each trial, then, three 70-mm lines spaced 20 mm apart for the three copies of each item, and to the right, a second box to end the trial.

Twenty words were chosen as items to copy (see Appendix). Ten were two-syllable words and ten three-syllable words. All were common singular nouns made up of eight letters, and none of them contained a schwa (in the French language, a schwa is an *e* at the end or in the middle of a word that is sometimes not pronounced, meaning that words have different numbers of syllables according to their modality; Chetail and Content, 2013). On the basis of the Lexique 3.5 French database (New et al., 2004; http://www.lexique.org), lexical frequency was controlled between the two lists, *t* < 1, as was the frequency of both the first syllable, *t* < 1, and the second syllable, *t* < 1. The frequency of the bigram at the syllable boundary was also controlled between the two lists, *t* < 1, as was the frequency of the bigrams before, *t* < 1, and after the boundary, *t*_(18_) = 2.02, *ns.*

### Procedure

Participants were tested individually. They were asked to write out the words displayed on the PC screen three times in a row. The participants triggered the beginning of the sequence by touching the start box on the left-hand side of the sheet of paper with their pen. The target word then appeared in the center of the PC screen. Next, the participants wrote it out three times, one on each line, and ended the trial by touching the finish box. In order to prevent participants from looking back at the target word, it disappeared as soon as they started to write. Moreover, in order to prevent lookbacks to previous copies, the experimenter hid each word as soon as it was written. These two processes were intended to obviate input processes between two copies of the same word, and ensure that only orthographic processes were engaged during the copy task.

Each participant wrote out the two 10-word sets in two successive conditions. First, they copied the words out without any time pressure. They were simply told to write the words out in their usual handwriting, as if they were writing a letter. Second, they were asked to write out the words “*as quickly as possible*,” and to move the pen as quickly as possible between each the three copies. The stated objective was to reduce the amount of time taken to produce all three copies. However, they were asked to maintain legibility. The intertrial pause was not speeded up, to enable participants to rest between each item. The two conditions were not counterbalanced across participants, as we assumed that starting with the time pressure instructions would influence the usual speed condition. The presentation of the 20 items was randomized. Each condition was preceded by four practice trials featuring different words from the experimental items. The entire testing session lasted ~25 min.

### Data analyses

The triple copy task enabled us to record three latencies–one before each copy. Because the first latency encompasses word recognition processes, making it difficult to disentangle the input and output effects, we only analyzed the duration of the second latency. For the letter measures, syllable boundary effects have been found both in the first copy (Kandel et al., 2006) and in the second (Sausset et al., 2012). We therefore restricted our analyses to the second copy (word duration, and measures at the single-letter level). The latency corresponded to the end of the first copy (i.e., word end + pressure = 0) and the onset of the second one (i.e., word beginning + pressure > 0). Measures at the letter level were extracted at the first syllable boundary for both the two- and three-syllable words. We examined the letter before the boundary (BB) (e.g., *r* in *mar.chand* [*seller*]), and the letter after the boundary (AB) (e.g., *c* in *mar.chand*). For these two letters, fluency, letter stroke duration and trajectory length were calculated as follows.

The data were first smoothed with a finite impulse response filter (Rabiner and Gold, 1975) with a 12 Hz cut-off frequency, using Ductus software (Guinet and Kandel, 2010). We then identified each letter of each word according to curvature maxima in the trajectory and velocity minima in the velocity profile, excluding the initial and final segments (Meulenbroek and Van Galen, 1990). Once each letter had been segmented, we extracted data on duration, length and fluency, again using Ductus. Since letters are compound and contain varying numbers of strokes (e.g., *c* has one stroke whereas *h* has three), the data values were normalized with respect to the number of strokes per letter. This normalization procedure was carried out in accordance with the letter segmentation analysis of cursive handwriting described by Meulenbroek and Van Galen (1990).

## Results

Words containing copying errors, as well as any data corrupted by a recording problem were eliminated from the analyses. Word durations and latencies greater or smaller than 2.5 standard deviations from a participant's and an item's conditional mean were discarded (2.8% for durations and 4.5% for latencies). At the letter level, data relating to incorrect or illegible letters were also eliminated from the analyses, as were data greater or smaller than 2.5 standard deviations from a participant's and an item's conditional mean for letter stroke duration and trajectory length. In addition, because the Eye and Pen software did not record data from the pen when it was not in contact with the graphics tablet, maximum velocity was sometimes reached when the pen was up, and the written trace only began when the movement was decelerating. In that case, because no velocity peak was recorded, the data pertaining to the corresponding letters were also discarded from the analyses. Altogether, 6.4% of the data on letter stroke duration, 6.6% of the data on letter stroke length, and 4.9% of the data on letter stroke fluency were discarded from the analyses.

Data were analyzed using generalized linear mixed models in SPSS 20.0, introducing random effects at the participant and item levels (Locker et al., 2007; Baayen et al., 2008). We used models with participant intercepts and item intercepts as random factors. For the word durations and latencies, the fixed effects included in the model were Syllable number (2 vs. 3), Writing condition (normal vs. time pressure), and the Syllable number x Writing condition interaction. For letter stroke duration, trajectory length and fluency, the model included Writing condition, Letter position (BB vs. AB) and the Writing condition x Letter position interaction.

### Word durations

Table 1 sets out the mean word durations in the normal handwriting and time pressure conditions as a function of syllable number. Word durations were shorter in the time pressure condition than in the normal handwriting one, *F*_(1, 774)_ = 471.47, *p* < 0.001. The effect of Syllable number was not significant, *F* < 1, nor was the Syllable number x Writing condition interaction, *F* < 1. Accordingly, time pressure affected word production by increasing writing speed.

**Table 1:** **Mean word durations (standard deviations) and mean latencies (standard deviations) in ms as a function of writing condition and syllable number**.

	**Syllable number**		
**Condition**	**2**	**3**	**All words**
**WORD DURATIONS**			
Normal	2709 (390)	2753 (385)	2731 (388)
Time pressure	2374 (354)	2385 (377)	2380 (365)
**LATENCIES**			
Normal	520 (103)	535 (100)	527 (102)
Time pressure	369 (69)	387 (78)	378 (74)

### Latencies

Table 1 sets out the mean latencies in the normal handwriting and time pressure conditions as a function of syllable number. Latencies were shorter in the time pressure condition than in the normal handwriting one, *F*_(1, 760)_ = 1018.10, *p* < 0.001. Latencies before two-syllable words were shorter than latencies before three-syllable ones, *F*_(1, 760)_ = 4.03, *p* < 0.05. The Syllable number x Writing condition interaction was not significant, *F* < 1. Thus, even though planning during latencies was shortened by time pressure, this did not affect syllable processing during these latencies.

### Letter stroke durations

The mean letter stroke durations as a function of position and writing condition are provided in Table 2. Mean stroke duration was longer in the normal handwriting condition than in the time pressure one, *F*_(1, 772)_ = 14.49, *p* < 0.001. Durations at AB were longer than those at BB, *F*_(1, 771)_ = 66.03, *p* < 0.001. The Letter position x Writing condition interaction was not significant, *F* < 1. In sum, letter stroke durations were longer for letters after a syllable boundary than for those before one, but this difference was not affected by time pressure, which simply increased the overall speed of letter writing.

**Table 2:** **Single-letter measures (means and standard deviations) as a function of writing condition and letter position in relation to the first syllable boundary**.

	**Letter position**		
	**Before boundary**	**After boundary**	**All letters**
**LETTER STROKE DURATION (in ms)**			
Normal	107 (45)	127 (61)	117 (55)
Time pressure	96 (37)	118 (57)	107 (50)
Both conditions	102 (42)	123 (59)	112 (52)
**LETTER STROKE TRAJECTORY (in mm)**			
Normal	6.86 (3.87)	10.23 (5.65)	8.57 (5.14)
Time pressure	6.76 (4.11)	10.08 (5.83)	8.42 (5.31)
Both conditions	6.81 (3.99)	10.16 (5.74)	8.50 (5.22)
**LETTER STROKE FLUENCY (in number of velocity peaks)**			
Normal	1.13 (0.47)	1.25 (0.67)	1.19 (0.58)
Time pressure	1.09 (0.57)	1.22 (0.46)	1.16 (0.65)
Both conditions	1.11 (0.46)	1.24 (0.66)	1.17 (0.56)

NB: a lower number of velocity peaks indicates a better fluency.

### Letter stroke trajectory length

Table 2 sets out the mean letter stroke trajectory lengths as a function of position and writing condition. The effect of Writing condition was not significant, *F* < 1. Trajectories were longer at AB than at BB, *F*_(1, 815)_ = 202.81, *p* < 0.001. The Letter position x Writing condition interaction was not significant, *F* < 1. These findings indicate that stroke trajectories were longer for letters after a syllable boundary than for those before one, and that this difference was not affected by time pressure.

### Letter stroke fluency

Table 2 sets out the letter stroke fluency means as a function of position and writing condition. Writing condition did not have a significant effect on fluency, *F*_(1, 964)_ = 1.78, *p* = 0.18. Results revealed a main effect of Letter position. Fluency was better at BB than at AB, *F*_(1, 963)_ = 21.32, *p* < 0.001. Writing condition and Letter position did not interact, *F* < 1. Thus, time pressure did not affect fluency, which was systematically better before the syllable boundary than after it.

## Discussion

The present study investigated the flexibility of the coordination of orthographic syllable planning and graphomotor execution during handwriting. This flexibility was investigated by manipulating the task demands and analyzing syllable processing. More specifically, writers were asked to copy out two- and three-syllable words three times in a row with and without time pressure. We analyzed handwriting latencies before the second copy, together with word duration, and the characteristics of the handwriting movements at the first syllable boundary. We reasoned that interactions between the time pressure condition and either syllable number (affecting latencies) or syllable boundary (affecting letter measures) would be evidence of flexibility in the coordination of handwriting processes according to task demands.

As expected, we found that time pressure had an impact both on latencies and on word durations. With time pressure, latencies were 28% shorter, and word durations 14% shorter. As far as handwriting movements are concerned, only letter stroke duration decreased under time pressure (10%). Letter stroke trajectory length and fluency were not affected. Thus, time pressure modified the temporal features of handwriting by increasing handwriting flow and shortening pauses (latencies). These findings are similar to those observed by Damian and Freeman (2008) for typing.

Again as expected, we found that the number of syllables in a word affected latencies. Latencies between two copies of the same word increased with the number of syllables (Lambert et al., 2008; Sausset et al., 2012). This is consistent with the idea that graphemic representations are structured on the syllable unit (Caramazza and Miceli, 1990; Ward and Romani, 2000). The fact that latencies were longer for longer words confirms that processing prior to execution concerns all the syllables in a word, and thus that orthographic processing takes place in advance, at least in part, when writers have to copy words in their usual handwriting. We also found an effect of time pressure at the syllable boundary: the duration and trajectory of the letter after a syllable boundary were longer than they were for the letter before a syllable boundary, and its writing was less fluent. This effect is usually interpreted as reflecting the cognitive demands of the programming of each syllable. Thus, the execution of the first syllable of a word is programmed beforehand, and that of the following syllables is programmed during the execution of their first letter. This has already been observed in adults writing in uppercase letters (Kandel et al., 2006; Álvarez et al., 2009), and in children (Kandel and Valdois, 2006a,b; Kandel and Soler, 2009; Kandel et al., 2009), but in the present experiment this syllable boundary effect was observed for the first time in adults using their usual handwriting. The fact that this effect is present even in adults, in whom execution is highly automatized, supports the idea that handwriting programming is organized on a syllabic basis rather than a letter one. The observation that syllable processing occurred both before and during handwriting was unexpected. However, further research is needed to investigate the nature of the processes underlying these two effects (which was not the focus of our study). It may be that the latency and syllable boundary effects do not reflect the same underlying processes. We interpret the effect of syllable number on latencies as reflecting the maintenance of orthographic sequences in the graphemic buffer, as evidenced by Lambert et al. (2008), but that the syllable boundary effect reflects graphomotor programming of the syllable.

Most importantly as far as this study is concerned, flexibility of syllable processing was not observed with time pressure. The effects of syllable number on latencies and syllable boundary on letter measures were similar regardless of whether or not any time pressure was exerted. Accordingly, even though time pressure influenced the temporal features of the writing movements, as shown by its effects on latencies, and led to a reduction in word and letter durations, it did not affect the actual coordination of the orthographic and graphomotor processes. All the syllables of the word were planned ahead of the transcription of the word, indicating that the orthographic processing in the graphemic buffer remained unchanged. In addition, the graphomotor execution of each syllable was then programmed at the syllable boundary to exactly the same extent in both conditions. Consequently, the time course of syllable processing does not appear to be flexible in handwriting, as argued by Damian and Freeman (2008; for similar results for speech, see Damian and Dumay, 2007).

This conclusion runs contrary, however, to that reached by Sausset et al. (2012), who demonstrated that orthographic planning cascades onto transcription when graphomotor constraints increase, and thus that planning consists in more or less advance syllable processing depending on task demands. We hypothesized that the changes in handwriting mode in their experiment had an impact not on the coordination of the processes, but on the processes themselves. Our reasoning was that when Sausset et al. asked their participants to write in uppercase letters, the graphomotor programming processes switched to a different programming unit. In the present study, therefore, instead of imposing different handwriting modes in a bid to constrain all the processes involved in the task, we chose to introduce a time pressure condition. Results showed that the coordination of orthographic and graphomotor processes remained the same regardless of condition. Thus, the difference between our results and those reported by Sausset et al. (2012) may stem from the nature of the task demands.

It is important to note that studies manipulating time pressure have also reported contradictory findings. For instance, time pressure sometimes causes planning to cascade onto execution (Kello et al., 2000), but not always (Damian, 2003; Damian and Dumay, 2007). This may be because the degree of time pressure imposed on participants varied between the different experiments. The consequences of time pressure do indeed change according to its intensity. For instance, high time pressure tends to have negative effects on behavior (e.g., effectiveness of decision making; Zakay and Wooler, 1984), whereas far from systematically impairing cognitive processing, moderate time pressure may even enhance it (e.g., creativity; Baer and Oldham, 2006). Moreover, writing under time pressure is a situation that is frequently encountered (Hartley, 2002; Connelly et al., 2005; Piolat et al., 2005), and our participants may thus have been familiar with this constraint.

In conclusion, we showed that time pressure does not influence the coordination of orthographic planning and graphomotor programming during handwriting. However, this does not necessarily mean that process coordination is inflexible. The fact that moderate time pressure does not have an impact on the cognitive processes subtending written production is not particularly surprising, given that writers often have to cope with time pressure. Further investigations are therefore needed to understand more clearly how different levels of time pressure affect the writing processes, by controlling the intensity of the pressure imposed on participants.

## Conflict of interest statement

The authors declare that the research was conducted in the absence of any commercial or financial relationships that could be construed as a potential conflict of interest.

## Appendix

**Table:** **Words used in the experiment, together with their corresponding word frequency (per million), syllable frequency, and the frequency of the bigrams around the orthographic syllable boundary (marked by a dot)**.

**2-syllable words**							**3-syllable words**						
	**Word frequency**	**Syllable frequency**		**Bigram frequency**				**Word frequency**	**Syllable frequency**		**Bigram frequency**		
		**First syllable**	**Second syllable**	***n − 1***	***n***	***n + 1***			**First syllable**	**Second syllable**	***n − 1***	***n***	***n + 1***
dis.cours	50.5	1128	160	4767	1130	4058	car.naval	5.7	205	785	15812	758	1346
mar.chand	27.9	1022	28	15812	2263	2301	pis.tolet	14.8	29	870	4767	4669	1775
pro.chain	28.6	2865	29	9806	761	2301	cri.minel	6.8	297	1798	3917	816	2443
froi.deur	7.6	30	363	3538	1121	2018	tri.bunal	15	214	214	3917	242	140
frac.tion	6.8	16	3909	1506	1174	3773	can.didat	2.8	86	1467	13340	4227	1592
fai.sceau	6.8	2732	7	12970	5640	254	cho.colat	30.6	1120	994	1413	761	755
clo.chard	5.3	121	11	1739	761	2301	pan.talon	57.9	123	1662	13340	9637	3386
por.trait	39.2	1430	232	7312	6796	3925	mu.sicien	5.9	385	1153	1006	2774	1871
vai.sseau	7.2	320	33	12970	5640	3639	pa.villon	20	4090	1353	20508	1485	1658
tour.ment	5.4	481	8497	7811	324	1792	vi.siteur	15.1	1805	1153	5188	4767	1871
*Mean*	18.5	1014	1327	7823	2561	2636		17.4	835	1145	8321	3014	1684
*SD*	16.7	1059	2791	5007	2461	1206		16.4	1274	464	6811	291	877

NB: n − 1 corresponds to the bigram preceding the boundary (e.g., is in dis.cours), n corresponds to the bigram straddling the boundary (e.g., sc in dis.cours), and n + 1 corresponds to the bigram following the boundary (e.g., co in dis.cours).
